# Increased risk of incident nasopharyngeal carcinoma with exposure to air pollution

**DOI:** 10.1371/journal.pone.0204568

**Published:** 2018-09-28

**Authors:** Hueng-Chuen Fan, Chiu-Ying Chen, Yi-Chao Hsu, Ruey-Hwang Chou, Chieh-Lin Jerry Teng, Chun-Hsiang Chiu, Chung Y. Hsu, Chih-Hsin Muo, Mei-Yin Chang, Kuang-Hsi Chang

**Affiliations:** 1 Department of Medical Research, Tungs' Taichung Metroharbor Hospital, Taichung, Taiwan; 2 Department of Pediatrics, Department of Medical Research, Tungs' Taichung Metroharbor Hospital, Taichung, Taiwan; 3 Department of Rehabilitation, Jen-Teh Junior College of Medicine, Nursing and Management, Miaoli, Taiwan; 4 Department of Public Health, China Medical University, Taichung, Taiwan; 5 Institute of Biomedical Sciences, Mackay Medical College, New Taipei, Taiwan; 6 Graduate Institute of Biomedical Sciences and Center for Molecular Medicine, China Medical University, Taichung, Taiwan; 7 Department of Biotechnology, Asia University, Taichung, Taiwan; 8 Division of Hematology/Medical Oncology, Department of Medicine, Taichung Veterans General Hospital, Taichung, Taiwan; 9 Department of Life Science, Tunghai University, Taichung, Taiwan; 10 Department of Medicine, Chung Shan Medical University, Taichung, Taiwan; 11 Division of Infectious Diseases and Tropical Medicine, Department of Internal Medicine, Tri-Service General Hospital, National Defense Medical Center, Taiwan; 12 Institute of Clinical Medicine, School of Medicine, National Yang-Ming University, Taiwan; 13 Graduate Institute of Biomedical Sciences, China Medical University, Taichung, Taiwan; 14 Management Office for Health Data, China Medical University Hospital, Taichung, Taiwan; 15 Department of Medical Laboratory Science and Biotechnology, School of Medical and Health Sciences, Fooyin University, Kaohsiung, Taiwan; Chinese University of Hong Kong, HONG KONG

## Abstract

**Background:**

Nasopharyngeal carcinoma (NPC) is a race-specific malignancy. The nasal cavity is the main entry point for air pollutants or poisonous gases into the human body. However, the risk of NPC in populations exposed to air pollution remains unknown.

**Methods:**

We combined data from the Taiwan Air Quality Monitoring Database (TAQMD) and the Longitudinal Health Insurance Database (LHID) to assess the risk of NPC in a population exposed to air pollution.

**Results:**

Multivariate analysis revealed positive trends for the association between the risk of NPC and exposure to air pollution. After adjusting for potential covariates, the risk of developing NPC increased with the increase in nitrogen dioxide (NO_2_) and fine particulate matter (PM_2.5_) exposure concentrations from 1.39 to 2.28 and 2.01 to 1.97, respectively, compared to the risks at the lowest concentration levels.

**Conclusions:**

We identified a significant risk of NPC in a population exposed to air pollution. However, this study had several limitations. Moreover, additional experimental and clinical studies on the associations between environmental factors and NPC risk are warranted.

## Introduction

Nasopharyngeal carcinoma (NPC) is a race-specific malignancy. It is endemic with a global incidence of <1 per 100,000 person-years in both men and women [1–3]. However, in Taiwan, its incidence ranges from 2.8 to 6.6 per 100,000 person-years, significantly higher than that in other countries [4, 5]. Moreover, among head and neck cancers, the incidence of NPC in Taiwanese women and men is in the highest and second-highest, respectively [6]. Although the complex etiology of NPC remains unclear, several epidemiological studies have suggested a significant association between NPC risk and a specific genotype, Epstein–Barr virus (EBV), and dietary habits [7–10]. In addition, exposure to active smoke, second-hand smoke, domestic fumes, and incense smoke increases the risk of NPC [5, 11, 12]. The nasal cavity is the main entry point for air pollutants and poisonous gases into the human body. However, the risk of NPC in populations exposed to air pollution remains unknown. Therefore, we conducted a longitudinal study by combining two national databases to assess the risk of NPC in a Taiwanese population exposed to air pollution.

## Materials and methods

### Data sources and study participant

We used two nationwide databases in order to clarify the risk of incident NPC in a Taiwanese population exposed to air pollution. The first, the Taiwan Air Quality Monitoring Database (TAQMD), was established and is maintained by the Taiwan Environmental Protection Agency. Data from this resource used in our analyses included records of the daily concentrations and distributions of two toxic air pollutants, sulfur dioxide (SO_2_) and nitrogen dioxide (NO_2_). The records of fine particulate matter (≤2.5 μm in diameter, PM_2.5_) between 1998 and 2010 from 74 ambient air quality-monitoring stations (AQMSs) were also included in the analyses. In northern Taiwan, there were 31 AQMSs, including 1 at Keelung City, 6 at Taipei City, 12 at New Taipei County, 6 at Taoyuan County, 3 at Hsinchu County and 3 at Miaoli County. In central Taiwan, the total number of AQMSs was 15 (3 at Nantou County, 6 at Taichung City, 3 at Changhua County, and 3 at Yunlin County). In southern Taiwan, there were 23 AQMSs, including 4 at Chiayi County, 4 at Tainan City, 12 at Kaohsiung City and 3 at Pingtung County. In eastern Taiwan, there were 5 AQMSs, including 2 at Yilan County, 1 at Hualien County and 2 at Taitung County. The location of the AQMSs was based on population density.

The second nationwide database is the National Health Insurance Research Database (NHIRD). In 1995, the Taiwan Bureau of National Health Insurance built the Longitudinal Health Insurance Database (LHID) to collect and maintain the medical records of >99% of Taiwanese residents from the beginning of 1996 to the end of 2010 [13]. We included patients with NPC from the Registry of Catastrophic Illnesses Patient Database (RCIPD) and the LHID, which are included in the NHIRD. The RCIPD was established to track patients with major or catastrophic illnesses including cancer, autoimmune diseases, and end-stage renal disease. In accordance with the Personal Information Protection Act, data from the insured were further recoded for identification before being made available to researchers. The Institutional Review Board of China Medical University, Taiwan, approved this study and waived the requirement for consent (CMUH-104-REC2-115). The diseases in the LHID are diagnosed in accordance with International Classification of Diseases, Ninth Revision, Clinical Modification (ICD-9-CM) codes.

On the basis of the living areas of each insured person and the locations of the AQMSs, the LHID and TAQMD were merged for analyses. The active areas of the insured were used to determine the site of the clinic or hospital with treatment records of acute infection of the upper respiratory tract (ICD-9-CM code 460). The inclusion criteria of this study were resident age more than 20 year and diagnosed without cancer from the beginning of 2000 to the date of NPC diagnosis, withdrawal from the LHID or RCIPD, or the end of 2011. Due to comprehensive concentration of PM_2.5_ was available from 2012; we excluded the resident with incomplete data on pollutants, age and sex.

We calculated the annual average concentration of each pollutant from 1998 to the year of observation, and quartiles were used to group the concentrations into four levels. To control for covariates, the confounders included in this study were participant age, participant gender, urbanization level, insurance fee level, occupational types, chronic sinusitis (CS, ICD-9-CM code 470), asthma (ICD-9-CM code 493), hypertension (HT, ICD-9-CM codes 401–405), chronic obstructive pulmonary disease (COPD, ICD-9-CM codes 490–496), hyperlipidemia (HL, ICD-9-CM code 272), diabetes mellitus (DM, ICD-9-CM code 250), EBV infection-related diseases (chronic active EBV infection, ICD-9-CM code 075; lymphoproliferative disease, ICD-9-CM code 238.7; Burkitt’s lymphoma, ICD-9-CM code 200.2; and Hodgkin’s disease, ICD-9-CM codes 201.0–201.9) and alcoholism (ICD-9-CM codes 303, 305·0, and V113).

### Statistical analytics

Categorical demographic data are shown as numbers and percentages. Continuous data are provided as means and standard deviations (SDs). We categorized pollutant concentrations into four levels according to quartiles, defined as the lowest, 2^nd^, 3^rd^ and highest concentration groups: SO_2_ concentration (lowest: <1,232.8 ppb; 2^nd^: 1,232.8–1,578.5 ppb; 3^rd^: 1,578.6–2,200.7 ppb; and highest: >2,200.7 ppb), NO_2_ concentration (lowest: <6,652.8 ppb; 2^nd^: 6,652.8–8,650.5 ppb; 3^rd^: 8,650.6–10,035.6 ppb; and highest: >10,035.6 ppb), and PM_2.5_ concentration (lowest: <10,759.7 μg/m^3^; 2^nd^: 10,759.7–12,161.4 μg/m^3^; 3^rd^: 12,161.5–15,056.4 μg/m^3^; and highest: >15,056.4 μg/m^3^).

The incidence density rates of NPC in person-years were calculated for each level. The hazard ratios (HRs) and 95% confidence intervals (CIs) of the risk of NPC at each level were analyzed by Cox proportional hazards regression. In multiple analysis, we controlled for covariates including age, gender, urbanization level, insurance fee level, occupational types, CS, asthma, HT, COPD, HL, DM, EBV infection-related diseases and alcoholism to estimate the risk of NPC according to air pollutant concentration levels.

## Results

We included a total of 162,797 participants (men: 71,397, 43.9%) with a mean age of 40.50 ± 14.63 years and a follow-up period of 11.70 ± 0.93 years. During the study period, 115 participants were diagnosed with NPC. Regarding urbanization level, approximately 34.5%, 32.5%, 16.9%, and 16.1% of participants lived in highly urbanized, moderately urbanized, boomtown, and other areas. The most frequently paid insurance fee was 14,400–21,000 NTD. The prevalence of CS, HT, COPD, asthma, HL, DM, and alcoholism was 0.1%, 13.7%, 18.5%, 1.8%, 8.3%, 4.3%, and 1.1%, respectively. The most common occupational type was white collar (58.9%). The annual average concentrations of SO_2_ (ppb), NO_2_ (ppb), and PM_2.5_ (μg/m^3^) were 1,819.7 ± 880.5, 8,240.1 ± 2,396.9, and 12,724.0 ± 3,197.2, respectively (Table 1).

**Table 1:** Distribution of the demographic data of the study participants.

n = 162,797		n	%
Age (years)	Mean ± SD	40.50	±14.63
Follow-up period (years)	Mean ± SD	11.70	±0.93
Sex (male)		71,397	(43.9)
NPC		115	(0.07)
Urbanization	Highly urbanized	56,122	(34.5)
	Moderately urbanized	52,904	(32.5)
	Boomtown	27,569	(16.9)
	Others	26,201	(16.1)
Insurance fee (NTD)	<14,400	25,193	(15.5)
	14,400–21,000	83,624	(51.4)
	>21,000	53,980	(33.2)
CS		131	(0.08)
HT		22,261	(13.7)
COPD		30,089	(18.5)
Asthma		2,991	(1.8)
HL		13,435	(8.3)
DM		7,031	(4.3)
EBV infection-related diseases	Chronic active EBV infection	30	(0.02)
	Lymphoproliferative disease	168	(0.10)
	Burkitt’s lymphoma	28	(0.02)
	Hodgkin’s disease	129	(0.08)
Alcoholism		1,786	(1.1)
Occupational types	White collar	95949	(58.9)
	Blue collar	46041	(28.3)
	Others	20807	(12.8)
Annual average of SO_2_ (ppb)	Mean ± SD	1,819.7	±880.5
Annual average of NO_2_ (ppb)	Mean ± SD	8,240.1	±2,396.9
Annual average of PM_2.5_ (μg/m^3^)	Mean ± SD	1,2724.0	±3,197.2

CS: chronic sinusitis

HT: hypertension

COPD: chronic obstructive pulmonary disease

HL: hyperlipidemia

DM: diabetes mellitus

SO_2_: sulfur dioxide

NO_2_: nitrogen dioxide

PM_2.5_: fine particulate matter (≤2·5 μ in diameter)

Table 2 shows the increased incidence of NPC with increased exposure to NO_2_ and PM_2.5_. The highest concentration groups had a significantly higher risk of developing NPC than the lowest concentration groups for both NO_2_ and PM_2.5_. The incidence of NPC increased with increased exposure to SO_2_, NO_2_, and PM_2.5_ concentrations from 6.14 to 6.25, 4.66 to 8.71, and 3.83 to 7.94 per 100,000 person-years, respectively.

**Table 2:** Incidence, incidence rate ratio, and adjusted hazard ratios of NPC for the four levels of air pollutant exposure.

			PY	n of NPC	IR	IRR	95%CI	
SO_2_	Lowest	<1,232.8	456120.8	28	6.14	1.00		
(ppb)	2^nd^	1,232.8–1,578.5	510617.9	28	5.48	0.89	0.53	1.50
	3^rd^	1,578.6–2,200.7	442669.0	28	6.33	1.03	0.61	1.74
	Highest	>2,200.7	496023.5	31	6.25	1.01	0.61	1.69
NO_2_	Lowest	<6,652.8	493064.7	23	4.66	1.00		
(ppb)	2^nd^	6,652.8–8,650.5	488702.1	29	5.93	1.27	0.74	2.20
	3^rd^	8,650.6–10,035.6	567734.2	32	5.64	1.21	0.71	2.06
	Highest	>10,035.6	355930.3	31	8.71	1.88	1.09	3.22
PM_2.5_	Lowest	<10,759.7	547806.2	21	3.83	1.00		
(μg/m^3^)	2^nd^	10,759.7–12,161.4	419530.1	32	7.63	1.99	1.15	3.45
	3^rd^	12,161.5–15,056.4	509768.9	28	5.49	1.43	0.81	2.52
	Highest	>15,056.4	428326.0	34	7.94	2.08	1.21	3.58

PY: person-years

n of NPC: number of patients with nasopharyngeal carcinoma

IR: incidence rate (per 100,000 person-years)

IRR: incidence rate ratio

After adjusting for the potential covariates, a significantly higher risk of developing NPC was observed with increased air pollution exposure. Regarding the NO_2_ concentrations, the adjusted HRs after exposure to the 2^nd^, 3^rd^, and highest levels were 1.39 (95% CI = 0.80–2.42), 1.47 (95% CI = 0.84–2.58), and 2.28 (95% CI = 1.29–4.01), respectively. Regarding the PM_2.5_ concentrations, the adjusted HRs after exposure to the 2^nd^, 3^rd^, and highest levels were 2.01 (95% CI = 1.16–3.49), 1.38 (95% CI = 0.78–2.45), and 1.97 (95% CI = 1.13–3.43), respectively. A positive trend was observed for NO_2_ in the multivariate analysis (p for trend = 0.006) (Table 3).

**Table 3:** Adjusted HR of NPC in the moderate and high concentration groups compared to that in the low concentration group.

		Adj. HR	95%CI		p for trend
SO_2_ (ppb)	Lowest	1.00			0.638
	2^nd^	0.94	0.55	1.58	
	3^rd^	1.13	0.66	1.94	
	Highest	1.07	0.63	1.82	
NO_2_ (ppb)	Lowest	1.00			0.006
	2^nd^	1.39	0.80	2.42	
	3^rd^	1.47	0.84	2.58	
	Highest	2.28	1.29	4.01	
PM_2.5_ (μg/m^3^)	Lowest	1.00			0.067
	2^nd^	2.01	1.16	3.49	
	3^rd^	1.38	0.78	2.45	
	Highest	1.97	1.13	3.43	

Adj. HR: adjusted hazard ratio in the multivariate analysis after adjusting for age, sex, insurance fee, urbanization, COPD, HT, HL, asthma, DM, CS, EBV infection-related diseases, occupational types, and alcoholism

SO_2_: sulfur dioxide

NO_2_: nitrogen dioxide

PM_2.5_: fine particulate matter (≤2.5 μm in diameter)

## Discussion

This longitudinal nationwide study combined data from the NHIRD and TAQMD national databases. This study enrolled 162,797 participants. During the study period, 115 participants were diagnosed with NPC. The NPC diagnosis was validated by laboratory data, imaging findings, and pathological findings for avoiding strict TBNHI fines. We observed a significantly higher risk of NPC in a Taiwanese population exposed to air pollution after adjusting for potential covariates.

Long-term exposure to solid or gaseous pollutants may induce tissue-specific inflammation and affect levels of inflammatory mediators such as interleukin 6 (IL-6) and tumor necrosis factor-α (TNF-α) [14–19]. TNF-α is crucial in NPC tumor development [20]. Furthermore, previous studies have shown that occupational exposure to acid vapor, particularly sulfuric acid, may play a role in NPC development [21, 22]. In 2015, a meta-analysis has found consistent evidence on the relationship between NO_2_ exposure and the risk of developing lung cancer [23]. Several investigators have indicated that n nitrite intake may also increase the risk of NPC development [24, 25]. Moreover, particulate matter has been classified as a group 1 carcinogen by the International Agency for Research on Cancer (IARC). This evidence explains the possible association between SO_2_, NO_2_, and PM_2.5_ exposure and NPC development.

In view of the long-term latency of cancer development, we classified the annual average concentration of each pollutant into four levels by quartiles. Furthermore, we observed that the pollutant concentrations slightly changed between 1998 and 2010. Although this method may not be accurate, it can be used to test our hypothesis.

Several biases may have occurred in this study. First, although this study had a long follow-up period, misclassification due to NPC development after the study period may have resulted in an underestimation of NPC risk. Second, the residential area of participants was determined on the basis of the location of the clinics or hospitals where they received treatment for acute respiratory infections. Therefore, healthy residents exposed to lower levels of air pollutants may have been excluded if they had no related medical records during the follow-up period, resulting in the underestimation of NPC risk. In the univariate analysis, we considered the impact of several covariates such as age, gender, urbanization level, insurance fee level, HT, COPD, HL, DM, CS, asthma, EBV infection-related diseases, occupational types and alcoholism, which are associated with tumor development [26–30]. In addition, several studies have reported that active smoking and alcohol consumption are associated with increased NPC risk [31, 32]. Nevertheless, the major limitations of the NHIRD is attributed to insufficient information on family history and lifestyle activities such as smoking and alcohol consumption. Despite these limitations, we performed multivariate analysis by adjusting for COPD and alcoholism as potential covariates. On the basis of NHIRD studies, COPD and alcoholism are considered proxy variables for smoking status and alcohol consumption, respectively [33–35]. Cigarette smoking has been directly linked to COPD [36]. Patients’ attitudes and drinking behaviors contribute to the diagnosis of alcoholism [37].

We conducted this longitudinal study to evaluate the risk of NPC in residents exposed to air pollution. Because of the low incidence and high loss to follow-up rate, similar studies assessing the relationship between environmental factors and cancer are relatively rare. Although the number of patients diagnosed with NPC was low (n = 115), the NPC incidence ranged from 3.83 to 8.71 per 100,000 person-years among the different pollutant groups. These findings concur with those of other NPC-related Asian studies [38, 39].

## Conclusion

The results of this study suggest a novel approach to identify the active areas and individual exposure levels in environmental-related studies. Furthermore, we used the diagnosis of COPD and alcoholism as the proxy factors of smoking and alcohol consumption.

In, summary, we observed a significantly increased NPC risk in a Taiwanese population exposed to air pollutants. However, this study had some limitations, including the lack of data on lifestyle activities and dietary habits in the NHIRD. Additional experimental and clinical studies evaluating the underlying mechanisms of toxic air pollutants and NPC are warranted.

## Abbreviations

NPC: Nasopharyngeal carcinoma
TAQMD: Taiwan Air Quality Monitoring Database
LHID: Longitudinal Health Insurance Database
SO_2_: Sulfur Dioxide
NO_2_: Nitrogen Dioxide
AQMSs: Ambient Air Quality-Monitoring Stations
NHIRD: National Health Insurance Research Database
RCIPD: Registry of Catastrophic Illnesses Patient Database
ICD-9-CM: International Classification of Diseases, Ninth Revision, Clinical Modification
EBV: Epstein–Barr virus
HT: Hypertension
HL: hyperlipidemia
COPD: Chronic Obstructive Pulmonary Disease
HL: Hyperlipidemia
DM: Diabetes Mellitus
SDs: Standard Deviations
CIs: Confidence Intervals
IL-6: Interleukin 6
TNF-α: Tumor Necrosis Factor-α.
